# Ullmann Reactions of Carbon Nanotubes—Advantageous and Unexplored Functionalization toward Tunable Surface Chemistry

**DOI:** 10.3390/nano9111619

**Published:** 2019-11-15

**Authors:** Anna Kolanowska, Anna Wioleta Kuziel, Rafał Grzegorz Jędrysiak, Maciej Krzywiecki, Emil Korczeniewski, Marek Wiśniewski, Artur Piotr Terzyk, Sławomir Boncel

**Affiliations:** 1 Department of Organic Chemistry, Bioorganic Chemistry and Biotechnology, Silesian University of Technology, Krzywoustego 4, 44-100 Gliwice, Poland; anna.kolanowska@polsl.pl (A.K.); Anna.W.Kuziel@gmail.com (A.W.K.); rafal.jedrysiak@polsl.pl (R.G.J.); 2 Institute of Physics—CSE, Silesian University of Technology, Konarskiego 22B, 44-100 Gliwice, Poland; maciej.krzywiecki@polsl.pl; 3 Faculty of Chemistry, Physicochemistry of Carbon Materials Research Group, Nicolaus Copernicus University in Toruń, Gagarin Street 7, 87-100 Toruń, Poland; e.korczeniewski@umk.pl (E.K.); Marek.Wisniewski@umk.pl (M.W.); aterzyk@chem.uni.torun.pl (A.P.T.)

**Keywords:** carbon nanotubes, functionalization, Ullmann reaction, chlorination, hydrophilization, electroconductive coatings

## Abstract

We demonstrate Ullmann-type reactions as novel and advantageous functionalization of carbon nanotubes (CNTs) toward tunable surface chemistry. The functionalization routes comprise *O*-, *N*-, and *C*-arylation of chlorinated CNTs. We confirm the versatility and efficiency of the reaction allowing functionalization degrees up to 3.5 mmol g^−1^ by applying both various nanotube substrates, i.e., single-wall (SWCNTs) and multi-wall CNTs (MWCNTs) of various chirality, geometry, and morphology as well as diverse Ullmann-type reagents: phenol, aniline, and iodobenzene. The reactivity of nanotubes was correlatable with the nanotube diameter and morphology revealing SWCNTs as the most reactive representatives. We have determined the optimized conditions of this two-step synthetic protocol as: (1) chlorination using iodine trichloride (ICl_3_), and (2) Ullmann-type reaction in the presence of: copper(I) iodide (CuI), 1,10-phenanthroline as chelating agent and caesium carbonate (Cs_2_CO_3_) as base. We have analyzed functionalized CNTs using a variety of techniques, i.e., scanning and transmission electron microscopy, energy dispersive spectroscopy, thermogravimetry, comprehensive Raman spectroscopy, and X-ray photoelectron spectroscopy. The analyses confirmed the purely covalent nature of those modifications at all stages. Eventually, we have proved the elaborated protocol as exceptionally tunable since it enabled us: (a) to synthesize superhydrophilic films from—the intrinsically hydrophobic—vertically aligned MWCNT arrays and (b) to produce printable highly electroconductive pastes of enhanced characteristics—as compared for non-modified and otherwise modified MWCNTs—for textronics.

## 1. Introduction

Carbon nanotubes (CNTs) constantly hold the promise of molecular ‘properties-by-design’ toward countless everyday-life applications from electronics to energy to biomedicine [1,2]. However, full and large-scale exploration of the nanotubes’ superb capabilities remains ‘enchanted’. The main obstacle is formation of entangled CNT three-dimensional networks built-up by means of van der Waals forces [3]. To fully exploit extraordinary properties of CNTs such as high tensile strength and stiffness [4,5], ballistic electroconductivity [6], high thermal conductivity [7], or tailorable optical properties, ‘debundling’ of CNT agglomerates should be the first and essential step [8,9,10]. Covalent modifications as more stable than adsorption tailor CNTs for the application-oriented post-modifications [11,12]. However, among the covalent methods, damage-inducing oxidation reactions dominate [13]. For example, treatment of CNTs with O_3_ [14], HNO_3_ [15], KMnO_4_ [16], HNO_3_/H_2_SO_4_ [17], K_2_Cr_2_O_7_/H_2_SO_4_ [18], piranha solution [19], or Fenton agent [20] not only introduces oxygen groups onto CNT surface, but also disrupts the structural integrity of CNTs by formation of new wall-defects [21]. Hence, many other covalent modifications have been sought, including addition and cycloaddition reactions, to reduce to a minimum the structural CNT defects thereafter [22,23].

By the chemist’s eye, CNTs could be considered as sp^2+ε^-hybridized π-conjugated macromolecules therefore susceptible to C=C addition, particularly at the ‘molecular hot-spots’, i.e., defects and metallic chiralities, and, to a lesser extent, C-H substitution at the edges and vacancies. In our previous works, we were frequently inspired by organic reactions proving them as transferrable to CNTs [24,25,26]. A decade ago, corannulene—the convex buckybowl fragment of a CNT tip—was functionalized by Gershoni–Poranne and co-workers via Ullmann-type reactions [27]. These reactions allow formation of C–N, C–C and C–O bonds from the aromatic substrates. Since corannulene is a building block of fullerenes and CNTs are ‘elongated’ fullerenes, we decided to study Ullmann-type reactions in the chemistry of CNTs. This is a completely unexplored attempt for CNT functionalization and the first report where chlorinated CNTs are used as *substrates* in the Ullmann-type reactions.

To date, CNTs have been indeed used *only* as a support in the heterogeneous catalysis (CuCl anchored to pre-functionalized CNTs) in the Ullmann coupling with aryl halides toward formation of C–N and C–O bonds. For example, stable and recyclable catalysts were synthesized from metformin- [28], thiosemicarbazide- [29] or CuO/Fe_2_O_3_-grafted [30] MWCNTs. Those results, although not exploring CNTs as the Ullmann-type substrate themselves, could promise their high reactivity at the interphase in heterogeneous reactions. In addition, if successful, this route of functionalization could open new areas of CNT chemistry leading to enhanced individualization of CNTs.

Based on the above premises, we formulated the aim of this study to establish the procedure for CNT functionalization via chlorination and subsequent Ullmann-type reaction for three general types of substrates—phenol, aniline, and iodobenzene. We comprehensively characterize the so-modified CNTs to confirm the successful and controllable functionalization and show this approach as versatile and efficient way to application-oriented CNTs of various morphology, i.e., aspect-ratio, chirality and wall-defectivity. In order to demonstrate usability of the reaction, we use the protocol to produce from MWCNTs modified by 1,3,5-trihydroxybenzene (THB): (1) superhydrophilic, highly aligned, vertical nanotube array (‘carpet’) from the intrinsically hydrophobic array, and (2) electroconductive paste (from isotropic nanotube powder) yielding an order of magnitude more conductive coating than from the paste prepared from pristine nanotubes.

## 2. Materials and Methods

### 2.1. Materials

Commercially available MWCNTs NC7000™ (9.6 nm diameter, 1.5 μm length, 90% purity, catalyst and catalyst support residues: ~9 wt.% Al_2_O_3_, ~1 wt.% of total Fe) were purchased from Nanocyl, Sambreville, Belgium [31]. Longer and thicker, here called ‘in-house’, MWCNTs were synthesized in our laboratory via catalytic chemical vapor deposition (c-CVD) in an STF1200 Tube Furnace (Across International, Livingston, NJ, USA) at 760 °C with a feedstock continuously injected to a preheater at 250 °C. The main carbon source was toluene while the catalyst precursor was ferrocene (5.5 wt.%). The carrier gas was argon with the flow-rate of 1.8 L/min. The feedstock was dosed at the rate 2.8 mL/h (heating zone 60 × 500 mm). Single-walled CNTs (SWCNTs) were purchased from TUBALL™ (Leudelange, Luxembourg) of predominant chirality (6,5). General characteristics of all CNTs used in the work are presented in Table 1.

Iodine trichloride (ICl_3_, pure), iodine monochloride (ICl, >95%), copper(I) iodide (CuI, 99.999%), 1,10-phenanthroline (>99%), caesium carbonate (99.9%), *N,N*-dimethylformamide (DMF, 99.8%) (Sigma Aldrich, Poznań, Poland), carbon tetrachloride (CCl_4_, pure), phenol (pure p.a.), aniline (pure), iodobenzene (pure), 1,3,5-trihydroxybenzene (95%) (POCh, Gliwice, Poland), *n*-hexane (pure p.a.), toluene (pure p.a.), acetonitrile (pure p.a.), and dimethyl sulfoxide (DMSO, pure p.a.) (Chempur, Piekary Śląskie, Poland) were used. Prior to use, aniline, phenol, toluene and DMF were purified according to well-established protocols [32].

### 2.2. Methods

#### 2.2.1. Synthesis of Chlorinated CNTs (CNT-Cl) 

CNTs (0.500 g) were introduced to ICl_3_ solution (0.1 M) in CCl_4_ (50 mL) and the reaction mixture was ultrasonicated for 1 h under argon [33]. Next, the suspension was magnetically stirred (1000 rpm) for 2 h. CNT–Cl were filtered off using a polytetrafluoroethylene (PTFE) filter (Merck Millipore; Warszawa, Poland; pore size 0.2 μm). Subsequently, CNT–Cl was rinsed thoroughly with *n*-hexane (ca. 150 mL) and water (ca. 100 mL) until no halogen was detected [reaction with silver(I) nitrate(V)] in the filtrate. To ensure the absence of the adsorbed halogens, CNT–Cl were additionally washed with boiling methanol (250 mL) in Soxhlet apparatus (12 h). CNT–Cl were dried to constant weight at 85 °C (12 h) yielding ~0.52 g of product (independently from the type of nanotubes used). For chlorination of (very carefully cut) in-house vertically aligned array of MWCNTs (‘MWCNT carpet’) (0.500 g), a modified procedure was used, i.e., the reaction mixture was magnetically stirred without the ultrasonication step for 12 h—to maintain the integrity of the ‘carpet’—yielding the chlorinated MWCNT array (0.510 g).

#### 2.2.2. Reaction of MWCNTs with Iodine Monochloride

MWCNTs (0.50 g) were introduced to CCl_4_ (100 mL). The reaction mixture was ultrasonicated for 1 h under argon [33]. Next, ICl (2.00 g) was added and the mixture was ultrasonicated at room temperature in the darkness for 24 h. The product was filtered off using a PTFE filter (Merck Millipore; Warszawa, Poland; pore size 0.2 μm). Subsequently, the product was rinsed thoroughly with CCl_4_ (ca. 100 mL), methanol (ca. 100 mL) and water (ca. 150 mL) until no halogen was detected [reaction with silver(I) nitrate(V)] in the filtrate. To ensure the absence of adsorbed halogens, CNT-Cl was additionally washed with boiling methanol (250 mL) in a Soxhlet apparatus (12 h). The modified CNTs were dried constant weight at 85 °C (12 h) yielding ~0.50 g of the product.

#### 2.2.3. Ullmann-Type Reactions of CNT-Cl

CNTs (0.100 g), CuI (0.030 g; 0.16 mmol), 1,10-phenanthroline (0.060 g; 0.33 mmol), Cs_2_CO_3_ (0.800 g; 2.5 mmol), substrate (phenol, aniline or iodobenzene, 5 mmol) and 50 mL of solvent (DMF, DMSO, toluene, or acetonitrile) were placed in a round-bottomed flask equipped with a reflux condenser ended with an argon-filled balloon. The mixture was heated (82 °C acetonitrile, 150 °C DMSO, 100 °C toluene, 120 °C DMF) for 48 h under stirring. After cooling down to room temperature, the product was filtered off using PTFE filter (Merck Millipore; Warszawa, Poland; pore size 0.2 μm). Modified MWCNTs were washed with methanol (ca. 50 mL) and *n*-hexane (ca. 50 mL) until a colorless filtrate was observed. The resulting material was dried at 85 °C (to constant weight) yielding products of weight from 0.112 g for DMF to 0.101 g for other solvents, respectively. The same procedure was used for 1,3,5-trihydroxybenzene (phloroglucinol, THB)) and in-house ‘MWCNT carpet’ as the substrates, and the protocol yielded 0.110 g of the superhydrophilic ‘MWCNT carpet’.

#### 2.2.4. Preparation of MWCNT-Based Electroconductive Pastes

The appropriate amount of CNT nanofiller was ultrasonicated in distilled water in an ultrasonic bath (Bandelin Sonorex Super RK106, 35 kHz, 480 W, Berlin, Germany) for 30 min. Next, the suspension was stirred using Silverson L5MA (Chesham, UK) (10,000 rpm). A commercially available acrylic base SX-150 was added in portions until the appropriate concentration was achieved. The resulting slurry was stirred for 1 h (10,000 rpm). The as-obtained paint was printed (using applicator Erichsen type BIRD, model 284, Hemer, Germany) on a cotton textile substrate, dried and fixated at 85 °C for 15 min. The printing-drying sequence was repeated until the predetermined layer thickness was applied.

### 2.3. Characterization

Scanning electron microscopy (SEM), energy-dispersive X-ray spectroscopy (EDS), high-resolution transmission electron microscopy (HRTEM), thermogravimetry (TGA), Raman spectroscopy, dispersibility, and wettability studies were used to characterize the changes in morphology and surface physicochemistry of functionalized CNTs. The elemental analysis of CNTs was performed using an EDS Thermo Noran System (London, UK) attached to the SIX HITACHI S-3400N SEM (Hemer, Germany). The samples were analyzed at 10 kV with a working distance of 4.5 mm. HRTEM images were taken using a transmission electron microscope F20X-TWIN (FEI-Tecnai) (Hillsboro, OR, USA) operated at 200 kV. One drop of sample dispersion in ethanol (96%, p.a.) was placed on a copper grid coated with an ultrathin amorphous carbon film, and then dried under ambient condition. TGA curves were recorded using a LINSEIS STA PT1600 thermobalance (Selb, Germany) in a heating rate of 10 °C/min under argon atmosphere. Raman spectra were obtained using a Senterra micro-Raman system (Bruker Optics, Billerica, MA, USA) at 532 nm (a green laser) by collecting 5 × 5 s spectra. XPS investigations were carried out with PREVAC EA15 hemispherical electron energy analyzer equipped with 2D-MCP detector (Rogów, Poland). The samples were irradiated with an X-ray source (PREVAC dual-anode XR-40B, Al-Kα line, energy 1486.60 eV). The system base pressure was 2 × 10^−8^ Pa. For the survey spectra, the scanning step was set to 0.9 eV with pass energy 200 eV, while for particular energy regions to 0.05 eV with pass energy 100 eV. All of the measurements were performed with the analyzer axis perpendicular to samples’ plane. The binding energy (B.E.) scale of the analyzer was calibrated to Au 4f_7/2_ (84.0 eV). Recorded data were fitted utilizing CASA XPS^®^ embedded algorithms and relative sensitivity factors. Shirley function was used for the background subtraction. The estimated uncertainty for components’ energy position determination was 0.1 eV. For determination of the water contact angle (WCA) the goniometer described by us previously was applied [34]. The volume of typical droplet was equal to 6 µL, at the temperature 25 ± 0.1 °C, the results from at least three measurements were averaged. The enthalpy of immersion was measured at the same temperature, using the calorimeter described previously [35], and the results were averaged from at least three measurements. Each sample was thermally desorbed at vacuum and the temperature of 120 °C for 4 h. SEM/EDS data were collected using a Quanta 3D FEG scanning electron microscope (FEI) and Leo 1430VP (Zeiss, Oberkochen, Germany) with BSE detector, and Quantax 200 for SEM (Billerica, MA, USA).

## 3. Results and Discussion

**Figure 1:**
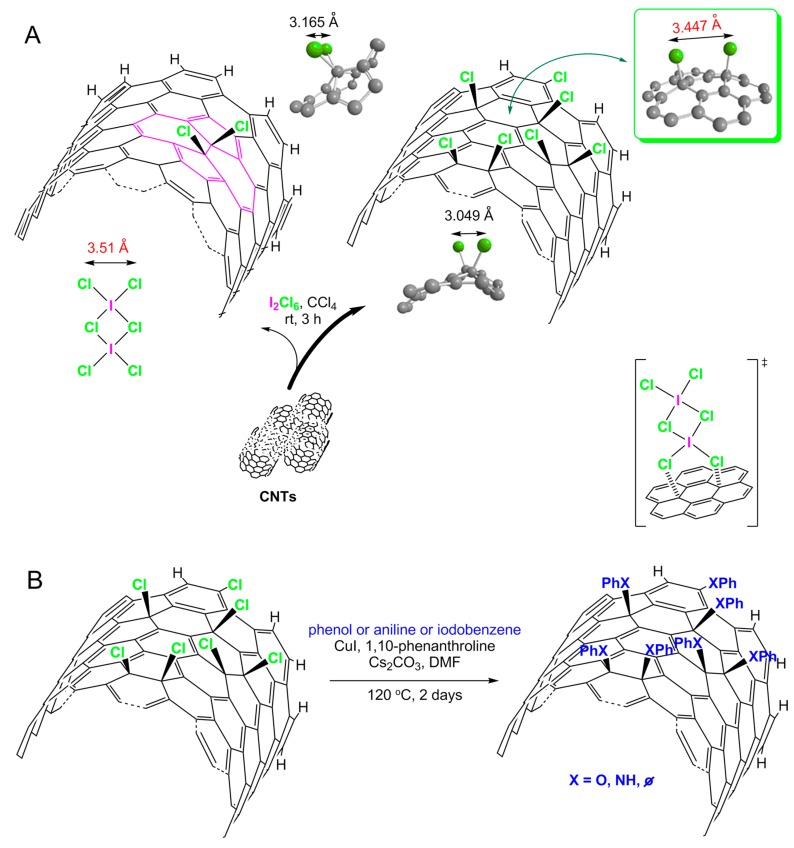
General scheme of the preferential sites of chlorination of carbon nanotubes (CNTs) by their treatment with ICl_3_ (**A**); optimized conditions for Ullmann-type reactions of CNTs (**B**); for clarity, only a fragment of the (outer) nanotube wall is shown; ∅ stands for an empty set, i.e., no atoms in the case of *C*-arylation. The figure was prepared on the basis of a hypothesis made by Abdelkader et al. [33].

The research was devoted to Ullmann-type reactions of CNTs, hence the initial substrate ought to be halogenated CNTs, here CNT–Cl (Figure 1). In order to select the optimal route of chlorination of CNTs, including safety and handling of the reagents, we decided to screen the ‘wet-chemistry’ methods. The optimum conditions were found for ICl_3_ in CCl_4_ as the chlorinating system [33]. This system could hypothetically enable preferential bis-1,4-addition over bis-1,2-addition (Figure 1A, thick reaction arrow). The regioselectivity results from ICl_3_ existing in solution as a dimer (I_2_Cl_6_) [36,37]. In this molecule, the distance between chlorine atoms in the Cl-I-Cl moiety equals the distance between chlorine atoms in the to–CNT bis-1,4-adduct, i.e., 3.5 Å. Other reactivity pathways, also indicated in Figure 1A (thin reaction arrow), would cover a reaction at the Stone–Wales defects as well as electrophilic substitution at the C-H termini and the wall gaps.

The copper-catalyzed Ullmann-type reaction was the next step (Figure 1B). Generally, this reaction is an attractive method for the formation of C-heteroatom and C–C bonds in organic synthesis [38]. This reaction of condensation is usually conducted at temperature as high as 200 °C, often in the presence of stoichiometric amounts of copper(I) catalyst and is reserved only for the active aryl halides [39]. However, a small amount of organic additive (e.g., 1,10-phenanthroline, diamines, aminoacids, diols, etc.) can be applied to increase solubility and stability of the copper(I) catalyst, and, at the same time, to allow performing the reaction under milder conditions. The copper (pre-)catalyst is prepared *in situ* from copper salt and the appropriate chelator [40]. Hence, the following stage was the reaction of CNT–Cl with three substrates: aniline, iodobenzene, and phenol for the formation C_CNT_–N, C_CNT_–C and C_CNT_–O bonds, respectively, in the presence of 1,10-phenanthroline and caesium carbonate (Cs_2_CO_3_) as a base (Figure 1B).

Since a classical Ullmann reaction was found as suffering from poor yields, we have investigated the effect of various solvents (toluene, acetonitrile, DMSO and DMF) on the effective coupling of substrates. Additionally, owing to the fact that the aryl halides show the reactivity order of I > Br > Cl > F, we have also investigated the effect of iodine presence on CNT surface on yield of the reaction. In order to evaluate it, we used ICl instead of ICl_3_ but eventually the latter emerged as the more active and more selective chlorinating agent.

**Figure 2:**
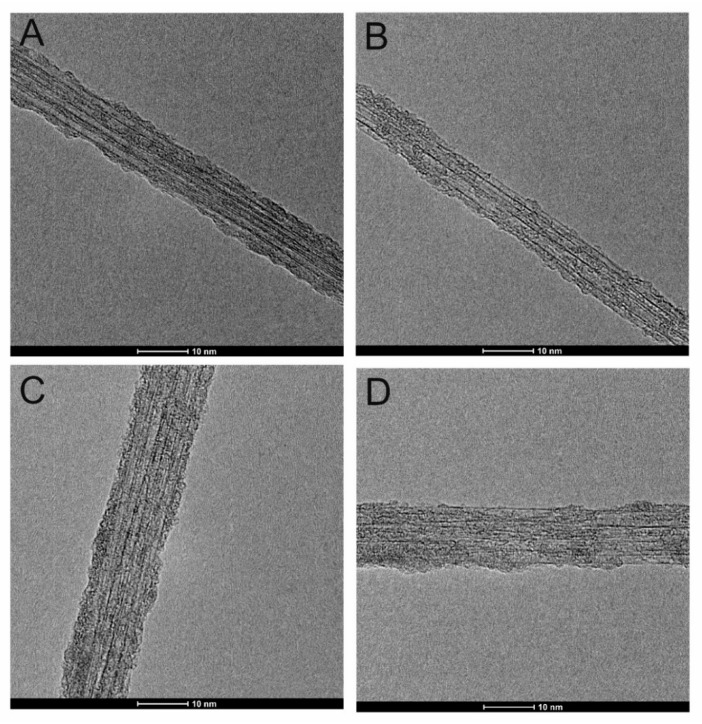
High-Resolution Transmission Microscope images (HRTEM) of chlorinated TUBALL™ single-walled carbon nanotubes (SWCNTs) by their treatment with: ICl_3_ (**A**), subjected to an Ullmann-type reaction with phenol (**B**), subjected to Ullmann-type reaction with iodobenzene (**C**), subjected to Ullmann-type reaction with aniline (**D**).

Figure 2 shows representative HRTEM images of chlorinated TUBALL™ SWCNTs (Figure 2A) and SWCNT–Cl subjected to Ullmann-type reactions with phenol (Figure 2B), iodobenzene (Figure 2C) and aniline (Figure 2D). One can observe that nanotubes form bundles. The influence of functionalization on the thickness of SWCNTs as well as the homogeneity of formed coating can be observed.

Generally, the layers covering nanotubes are not uniform and the functionalization takes place locally, starting probably at the wall defects. In some cases (Figure S1), we also observed ‘debundling’ of the single tubes.

In order to determine the functionalization degrees, we performed TGA measurements in argon atmosphere, i.e., under pyrolytic conditions (Figure S2, Supplementary Materials). Assuming the weight loss above 300 °C could be ascribed to disruption of the covalent bonds, we quantified functionalization degrees (mmol g^−1^ CNTs) (Table 2). The functionalization degrees were determined by a projection of Derivative Thermogravimetry (DTG) peak onsets and offsets onto the corresponding TGA curves and readout of the corresponding weight differences between the marked points.

As can be seen, the highest functionalization degrees were observed for chlorinated TUBALL™ SWCNTs and Nanocyl NC7000™ MWCNTs—both achieved for the reactions performed in DMF. Contrarily, the lowest functionalization degrees were found for chlorinated Nanocyl™ MWCNTs in DMSO and in-house MWCNTs in DMSO and acetonitrile. Chlorinated SWCNTs and shorter/thinner as well as more defective MWCNTs—in terms of number of crystallographic wall-defects—emerged as the more reactive species in the Ullmann-type reactions. Additionally, reactivity of CNT-Cl could be correlated with dispersibility of CNTs in the tested solvents, indeed proving DMF as the most promising solvent for both pristine and chlorinated nanotubes [41,42]. It can be also readily noted that ICl_3_ emerged as the superior reagent than ICl for halogenation of CNTs—functionalization degree for the former was ca. five times lower than for ICl_3_. Therefore, even though in the case of ICl more iodine atoms calculated per chlorine were introduced on the CNT surface, their effects on the further transformations were essentially limited. This behavior could be probably connected with the synergic effect between CNT surface and the structure I_2_Cl_6_ [43]. Furthermore, the side-product of this reaction, i.e., I_2_Cl_4_ decomposes to ICl and ICl_3_ [33], hence partly regenerating the chlorinating agent. 

EDS analysis (Figure S3, Supplementary Materials) was conducted on six zones of the pristine and functionalized Nanocyl NC7000™ MWCNTs as the most representative and large-scale applicable nanotubes. The main elements and their concentrations found in the statistically valid analyses are listed in Table 3. As seen, the element of the highest atomic concentration in all of the MWCNT samples, as expected, was found to be carbon. It was accompanied by corresponding and significant contents of chlorine, oxygen, or nitrogen atoms after the covalent modifications. Importantly, after ICl_3_ treatment, the samples contained carbon, chlorine and only very small amounts of iodine atoms while after reactions with ICl atomic—both total and relative I/Cl concentrations—were different, i.e., lower and in favor of iodine. Moreover, in the latter case, the sample contained aluminum of 1.43 at.% since ICl as the halogenating agent was found to be incapable to fully transform aluminum residues into volatile side-products. These results agree with the expected mechanism of chlorination using ICl_3_. Accordingly, the *in situ* released chlorine could remove the catalyst nanoparticles and amorphous carbon contaminations (residuals from the industrial c-CVD synthesis) [44]. Hence, CNTs can be synchronically functionalized and purified via ICl_3_ treatment. Importantly, those small amounts of aluminum were detected only in the ICl-functionalized CNTs. Ullmann-type reactions of chlorinated MWCNTs with phenol, aniline, and iodobenzene resulted in the presence of oxygen, nitrogen and carbon atoms on the CNT surface replacing the chlorine atoms. However, conversion of chlorine into these elements was not complete and small amount of chlorine was detectable. Atomic concentration of chlorine was found to be in a good correlation with the results calculated from TGA curves. For *O*-, *N*- and *C*-arylated MWCNTs, a higher atomic concentration of oxygen, nitrogen and carbon was found, respectively, confirming them all as successful covalent functionalizations of similar yields.

**Figure 3:**
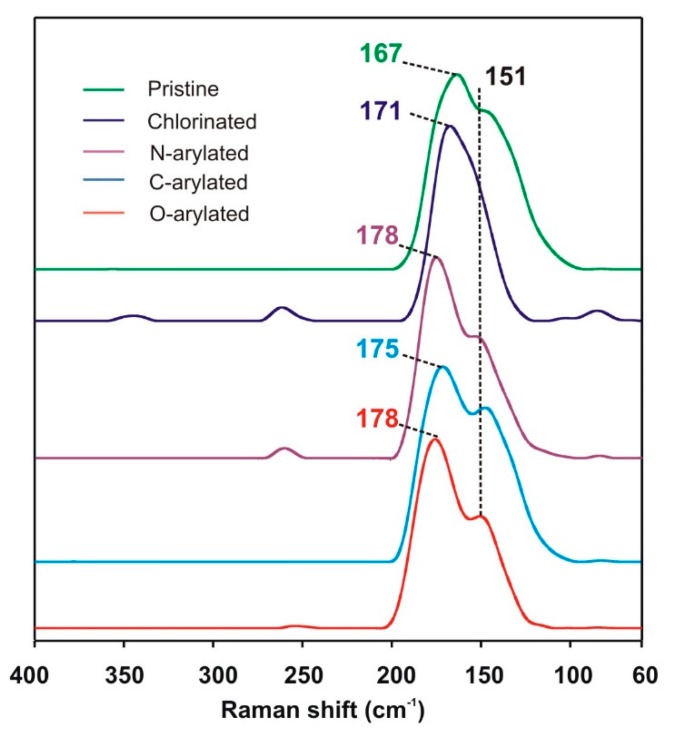
The Radial Breathing Mode (RBM) region of pristine and functionalized SWCNTs.

To look into the in-depth molecular structure of functionalized SWCNTs, Raman spectra were acquired. Figure S4 represents Raman spectra of TUBALL™ SWCNTs: pristine, chlorinated, *O*-arylated, *N*-arylated, and *C*-arylated. The most important three features were found: (1) the sp^3^ C-atoms, D-band at 1335–1341 cm^−1^, (2) tangential G-band at 1540–1620 cm^−1^ related to the graphitic structure of CNTs, and (3) radial breathing mode (RBM) at 100–200 cm^−1^, which corresponds to the nanotube radial contraction/expansion and is correlatable with the SWCNT diameter (Figure 3). G’-bands at 2500–2700 cm^−1^ could be also found. The experimental relationship d = 248/υ, where υ is the Raman shift of the RBM in cm^−1^, allows for determining the SWCNT diameter (d). The RBM analysis of initial SWCNT sample shows a dual mode distribution of SWCNTs with diameters of 1.48 and 1.64 nm calculated respectively from the bands at 167 and 151 cm^−1^. 

Upon functionalization, distribution of the nanotube average diameters changed. Firstly, after chlorination, the diameter peak was shifted to 171 cm^−1^ (corresponding to d = 1.45 nm), which could be caused by an increase in the pyramidalization angle due to electron withdrawing from the tubes caused by the attachment of chlorine atoms. Furthermore, shifts of RBMs, due to the decrease in CNT electron density, were observed when chlorinated tubes were arylated, meaning that number of atoms, disrupted in the hexagonal framework, increased. Additionally, the signal originating from the larger tubes at 151 cm^−1^ remained unchanged, confirming higher reactivity of chlorinated SWCNTs of the smaller diameter. 

**Figure 4:**
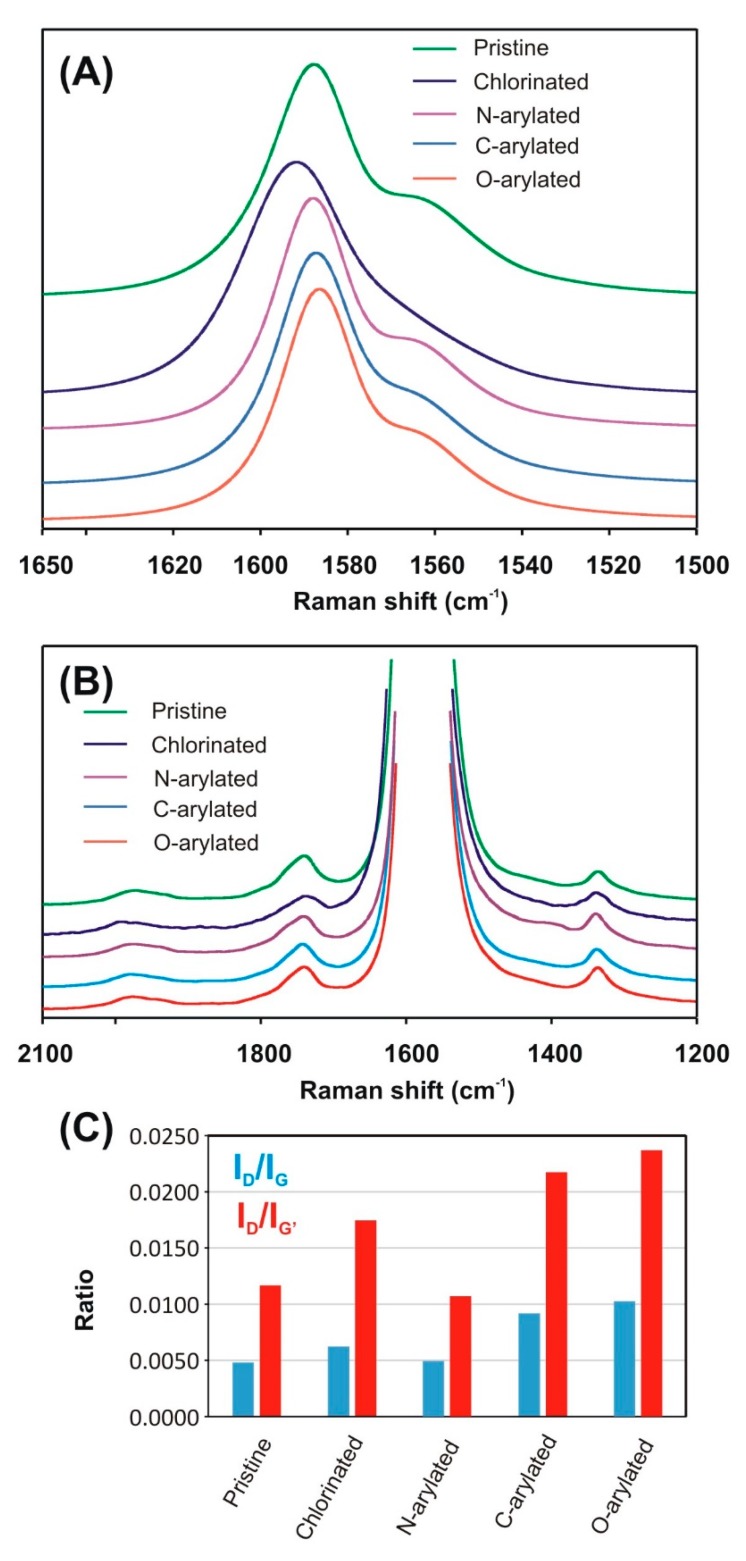
The G-band (**A**) and D-band (**B**) region of SWCNT samples; (**C**) I_D_/I_G_ and I_D_/I_G’_ ratios presented as blue and red bars, respectively.

Furthermore, very interesting spectral changes were observed in the D- and G-regions (Figure 4). Thus, while the G-band changed its position and intensity upon functionalization (Figure 4A), the D-band did not (Figure 4B). It should be noted that the D-band is typically qualitatively and quantitatively related to the sp^2^-C-defects, i.e., number of sp^3^-C-atoms. The G-band in the spectra of SWCNT samples revealed signals at 1565 cm^−1^ and at 1588 or 1599 cm^−1^ (Figure 4).

Hence, the I_D_/I_G_ ratio could be taken as a measure of sp^3^-defect concentration [45]. The defects here mean the C-atoms which underwent addition and the subsequent reaction. The I_D_/I_G_ ratio (Figure 4C) increased from ca. 5×10^–3^ (pristine SWCNTs) to even 0.01 (*O*-arylated SWCNTs), indicating conversion of sp^2^ to sp^3^-C atoms in the functionalized SWCNTs and confirming the observations from the RBM region and TGA results. The other ratio used for characterization of SWCNTs is I_D_/I_G’_ (Figure 4C). The small I_D_/I_G’_ ratio is the evidence of high crystallographic order in the nanotube walls [45]. Therefore, a high level of crystallographic perfection could be assigned to pristine SWCNTs (I_D_/I_G’_ ca. 0.012), while, after functionalization, I_D_/I_G’_ increased up to 0.024 for *O*-arylated SWCNTs.

**Figure 5:**
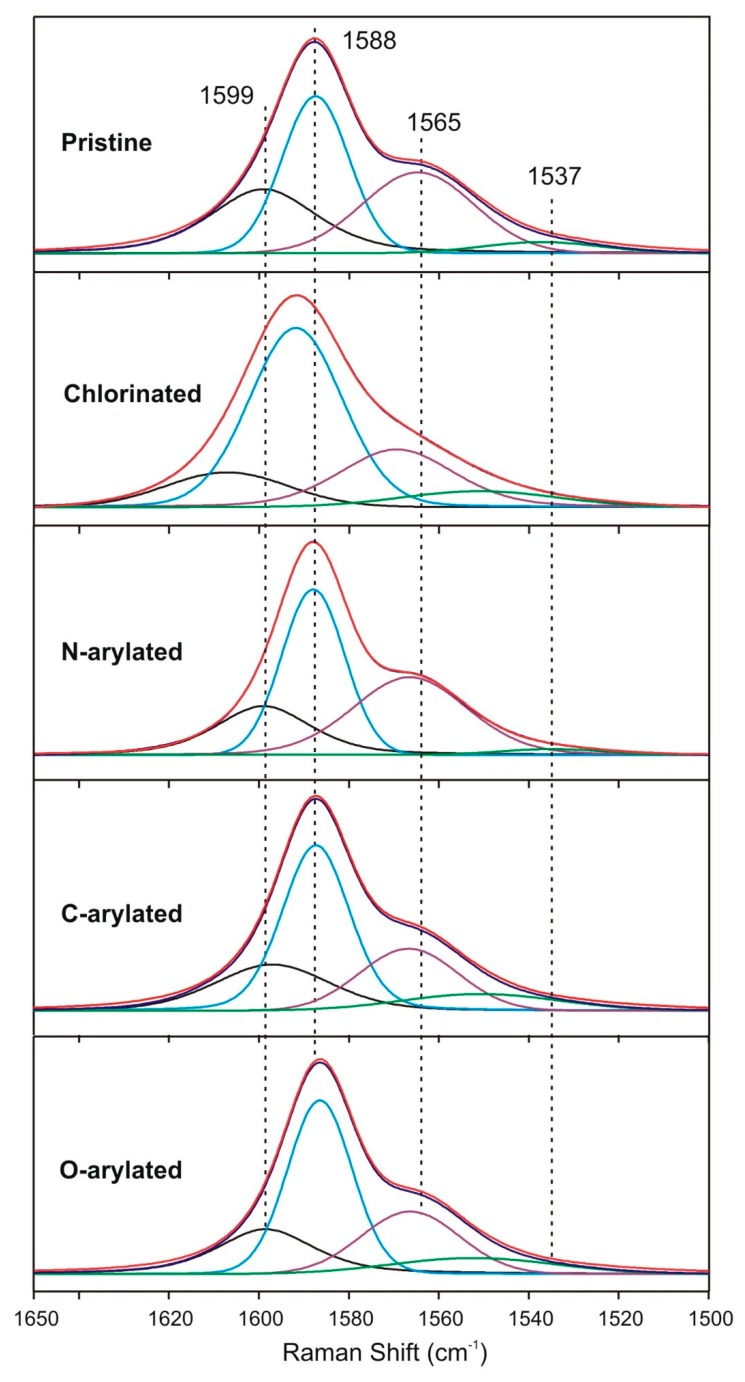
The G-band range after deconvolution for pristine and functionalized single-walled carbon nanotubes (SWCNTs).

Nevertheless, not only intensity, but also shifts of the D- and G-bands provide information on functionalization of CNTs. To track changes in the G-band positions, we have carried out a line-shape analysis in order to minimize the number of independent fitting parameters. For all tested SWCNTs, we have fitted the region from 1500 to 1650 cm^−1^ with the sum of four independent components and the results—as fitted Raman spectra of G-band of pristine and modified SWCNTs—are presented in Figure 5. 

Therefore, while the positions of D-band remained practically unchanged for all SWCNT samples, all the three G-bands for chlorinated SWCNTs were shifted to higher frequencies due to an electron-withdrawing effect of chlorine atoms (*p*-type doping). In the case of arylated SWCNTs, due to an electron-donating effect (*n*-type doping), the signals were shifted to lower frequencies at 1565 and 1588 cm^−1^. 

A more careful analysis of the two G-band features showed that the G^+^ and G^–^ features were both composed of two peaks of different symmetries. While the two most intense peaks (G^+^ and G^−^) at ∼1588 and ∼1565 cm^−1^, respectively, would arise from phonons with A and E_1_ symmetries, the lower intensity features at ∼1537 and ∼1599 cm^−1^ would be associated with the E_2_ symmetry phonons [46]. Moreover, the G^+^-feature is associated with carbon atom vibrations along the nanotube axis (LO phonon mode), and its frequency is sensitive to charge transfer from dopant additions to SWCNTs. The G^–^ feature is associated with vibrations of carbon atoms along circumferential direction of the SWCNTs (TO phonon mode), and its line-shape is highly sensitive to whether the SWCNTs is metallic or semiconducting [46].

After chlorination, all G^–^ and G^+^ signals were shifted ca. 10 cm^−1^ to the higher wavenumbers. Contrarily, arylation caused that G^+^-bands were downshifted to their initial positions, while both G^–^-bands remained upshifted several cm^−1^. *N*–, *O*–, and *C*-aryl as well as chloro groups exhibit a dual and opposite character in terms of deforming electron density at the neighboring aromatic fragments, i.e., electron-withdrawing by induction and electron-donating by mesomerism. These characteristics are revealed in the Raman spectra of G^+^-bands—where, for chlorinated SWCNT signals, they were shifted to higher frequencies as the consequence of electron transfer from the π-states of SWCNTs. This behavior shows domination of the inductive effect. It should be emphasized that this net effect was not observed for SWCNTs after *O*–, *C*–, and *N*-arylation confirming negligible changes in the electron density at the centers of functionalization. After chlorination, due to the less efficient overlap of CNT π-electrons with chlorine *d*-electrons, the domination of the inductive over the mesomeric effect was more pronounced. Importantly, the D-band remained unchanged independently on the reaction type. It means that disordered C-atoms did not react proving chemoselectivity, i.e., enhanced reactivity, of aromatic sp^2^- over sp^3^-carbon atoms in the Ullmann type reactions of CNTs. 

**Figure 6:**
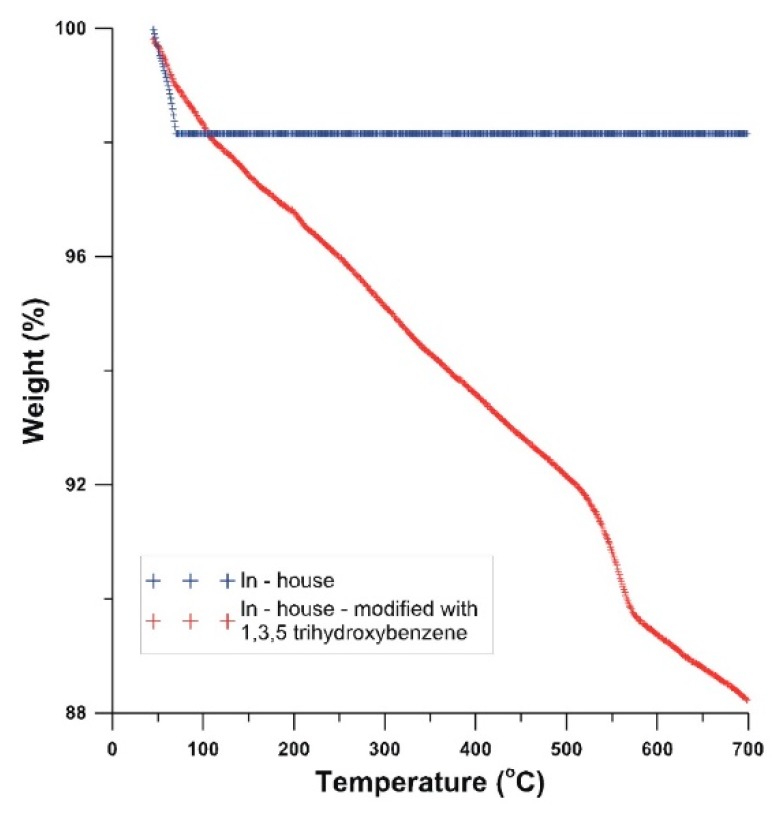
Thermogravimetric (TG) curve for 1,3,5-trihydroxybenzene (THB) modified in-house vs. a pristine MWCNT array sample.

Additionally, in order to prove versatility, efficiency, and tunability of surface chemistry of Ullmann-type reactions of CNT–Cl, we used the optimized reaction conditions for controlled hydrophilization of intrinsically hydrophobic vertically aligned MWCNT arrays (‘MWCNT carpets’) using 1,3,5-trihydroxybenzene (THB) as the coupling substrate. For this substrate, functionalization degree—as determined by TGA—was equal to 0.96 mmol g^−1^ CNTs (Figure 6). Its analysis will be discussed further in the part of the manuscript concerning hydrophilization of CNT arrays.

**Figure 7:**
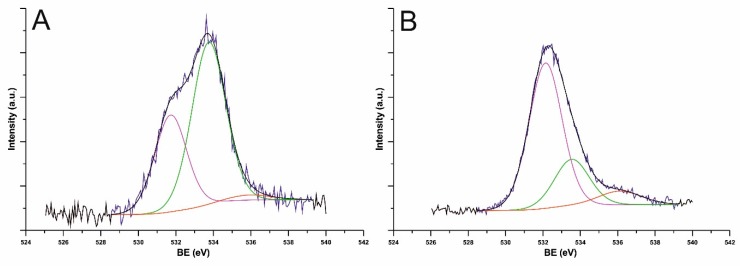
O-1s region X-ray photoelectron spectroscopy (XPS) signals and deconvoluted peaks for *O*-arylated Nanocyl NC7000^TM^ MWCNTs modified via reaction of chlorinated nanotubes with phenol (**A**) and 1,3,5-trihydroxybenzene (**B**).

Figure 7 shows XPS spectra obtained in the O-1s bonding energy (BE) region for *O*-arylated MWCNTs (Figure 7A) and modified with 1,3,5-trihydroxybenzene (Figure 7B). There are three key peaks at 532, 534, and 536 eV. The peak related to the C–O bond is observed at BE of 534 eV, while the one attributable to C–OH bonds appear at 532 nm. The weak peak at 536 eV can be assigned generally with the physisorbed oxygen molecules.

**Figure 8:**
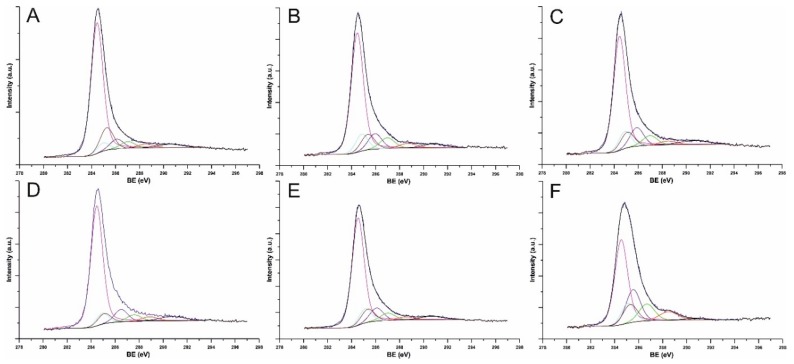
High resolution X-ray photoelectron spectroscopy (XPS) of Nanocyl NC7000^TM^ MWCNTs obtained in the C 1s bonding energy region for: halogenated CNTs with ICl_3_ (**A**), halogenated CNTs using ICl (**B**), *O*-arylated CNTs (**C**), *N*-arylated CNTs (**D**), *C*-arylated CNTs (**E**), modified with 1,3,5-trihydroxybenzene (THB) (**F**).

Figure 8 shows the peaks of photoemission for C-1s for covalently modified Nanocyl MWCNTs. The main peak for the sp^2+ε^-carbon atoms such as in CNTs is located at BE ~285 eV. Because of the high concentration of sp^2+ε^-carbon atoms, the peaks are broad with a longer asymmetric tail toward higher BE-values. As the effect of functionalization, the concentration of sp^3^-carbon atoms increased, which resulted in the symmetric peak at 285.5 eV. The peaks corresponding to C-O (286.7 eV) and C=O (287.8 eV) bonds could be assigned to adsorbed oxygen and carbon dioxide, respectively. Further, the peaks at 284.8 and 285.2 (low intensity) correspond to C–I and C–Cl bonds on the CNT surface. XPS analysis of the substrates, i.e., ICl- and ICl_3_-chlorinated MWCNTs was also performed. Therein, inter alia the Cl-2p energy regions were analyzed (Figure S5). The Cl 2p regions exhibit one highly symmetrical component with its spin-orbit splitting counterpart representing Cl–C bonding at ~200 eV.

**Figure 9:**
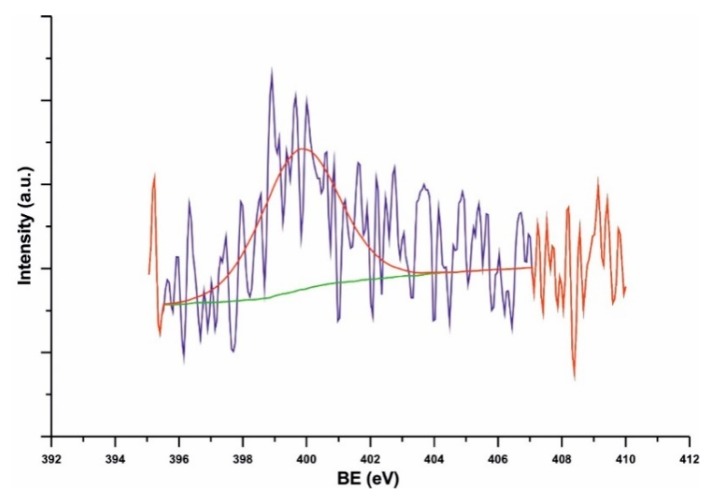
High resolution X-ray photoelectron spectroscopy (XPS) of Nanocyl NC7000^TM^ MWCNTs obtained in the N 1s bonding energy region for *N*-arylated MWCNTs.

Figure 9 shows the XPS spectra of *N*-arylated Nanocyl NC7000™ MWCNTs. The occurrence of N 1s peak at 400 eV indicated the presence of N–H groups in the sample while the presence of C–N bonds was demonstrated in the region of C 1s peak at 286 eV.

**Figure 10:**
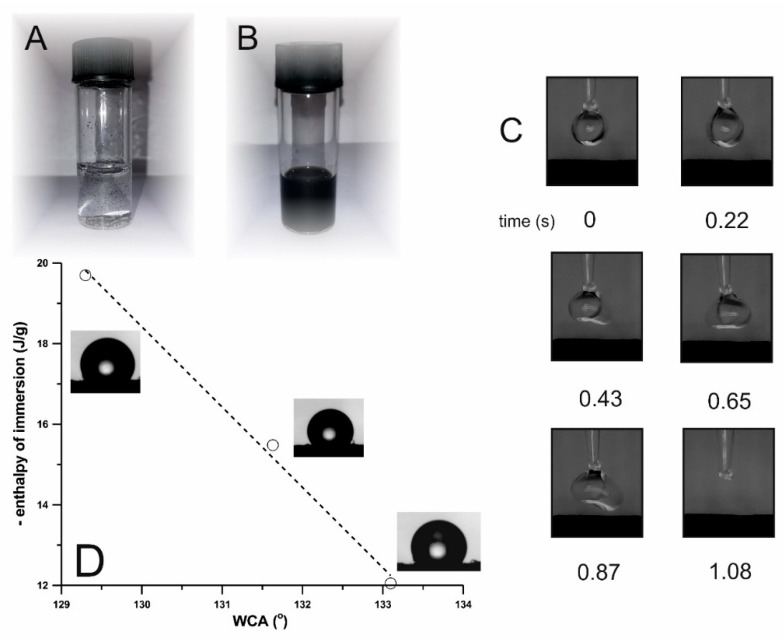
Aqueous dispersions of in-house MWCNT array tiny flakes: pristine (**A**) and covalently modified by Ullmann-type reaction of chlorinated arrays with 1,3,5-trihydroxybenzene—identical weight and no ultrasonication applied (**B**); wetting dynamics of 1,3,5-trihydroxybenzene (THB) covalently modified in-house ‘MWCNT carpet’—snapshots of photographs in the timescale of seconds for a drop released from a goniometer (**C**); the correlation between wetting contact angle (WCA) and the enthalpy of immersion for three samples: pristine, blank experiment (no catalyst in the Ullmann reaction) and non-covalently treated with 1,3,5-trihydroxybenzene in-house vertically aligned MWCNT array (**D**).

Concerning the reaction of chlorinated MWCNT arrays with hydrophilic THB (Figure 10), the presence of multiple hydroxyl groups on the final MWCNT surfaces resulted in their immediate water-dispersibility (Figure 10B) as opposed for completely indispersible, hydrophobic pristine MWCNTs (Figure 10A).

Additionally, for the surface-wetting characterization, WCA were measured for vertically aligned MWCNT arrays: pristine (as-made), blank experiment (all reactants and conditions the same but reaction performed in the absence of catalyst), non-covalently, and covalently treated chlorinated nanotubes with phloroglucinol. For the pristine MWCNT array, the contact angle was found to be equal to 129.3°. After chlorination, as well as after an Ullmann-type reaction, immediately after placing the droplet on the top of the MWCNT array (‘carpet’/‘forest’), the droplet was drawn into the inter-tube space, which could be actually considered as a superhydrophilic effect with the contact angle close to 0° (though time of infiltration for chlorinated array was just above 1 s). This behavior is shown for one of the systems in Figure 10C presenting the timeline for the wettability test. As can be seen, a water droplet soaked into the carpet within nearly 0.5 s. The hydroxyl groups introduced onto the MWCNT surface caused a change in the contact angle due to formation of the 3D-network of hydrogen bonds and hence the superior wettability. For non-covalently modified (blank) samples, MWCNT carpet was heated in 1,3,5-trihydroxybenzene DMF solution (identical procedure for Ullmann-type functionalization but performed in the absence of the catalyst and base) yielding WCA equal to 131.6°, while, for the carpet saturated with DMF solution by a Pasteur pipette, WCA was 133.1°. Finally, Figure 10D shows that, for the samples where the measurement of WCA was possible, we observed the occurrence of linear type relationship between the WCA values and the value of enthalpy of immersion in water, i.e., with the rise in hydrophobicity the WCA increased. Simultaneously (in contrast to chlorinated and Ullmann reaction treated chlorinated arrays (‘forests’), all three surfaces were hydrophobic (WCA > 90°).

Finally, it is interesting to mention that the modification applied in this study led also to some structural changes of the studied nanotube forests. SEM/EDX analyses led to the conclusion that, after chlorination, the tubes at the surface are more vertically arranged than the tubes at the bottom part of the forest (Figure S6A,B). In contrast, for nanotubes after an Ullmann-type reaction, we observed densification of the nanotubes at the top carpet surface (Figure S6C,D). Finally, for the remaining nanotubes, no strong structural changes were observed. Typical EDS data for the forest functionalized using an Ullmann-type reaction are collected in Figure S7. One can observe the densification of nanotubes at the top of the forest, and the even distribution of chlorine in all of the forest layers. The presence of small amounts of chlorine confirms <100% conversion of chlorine atoms in Ullmann-type reactions.

Due to the promising results of dispersibility in water, we have also prepared the electroconductive paste based on commercial resin containing isotropically dispersed 5 wt.% of in-house MWCNTs modified with THB and applied it on cotton (layer thickness—200 µm). The surface electrical resistivity—as measured by our recently published methodology [47]—was 490 ± 30 Ω sq^−1^. Importantly, this value was found to be more than two times lower than for in-house MWCNTs modified with sodium dodecylsulphonate (SDS), where solely the aspect ratio, at a medium dispersibility of nanotubes, was the key factor governing electroconductivity of coatings for textronic applications.

## 4. Conclusions

Ullmann-type reactions were found to be an effective and versatile route for CNT functionalization. The main advantages of the synthetic protocols—elaborated for both powder and thick film macro-assemblies—represent a universal methodology for the formation of C_CNT_–OAr, C_CNT_–NH–Ar and C_CNT_–(C)Ar bonds, predictable reactivity of CNTs of various morphology and tunability of the CNTs’ properties by the selection of the substrates. From the applicability point-of-view, the protocol allows for producing CNTs of tunable hydrophilicity and enhanced—by higher nanotube dispersibility—electroconductivity of coatings for textronics. Moreover, the protocol constitutes an important premise for functionalization of CNTs via C–P and C–S bonds in the nearest future. For example, in the Ullmann-type reactions, aryl iodides, bromides, chlorides, and tosylates were used as the substrates while *S*-nucleophiles were thiophenols, thiourea, aliphatic, and aromatic thiols. Similarly to our procedure, copper halide [48], Cs_2_CO_3_ (as a base) [49] and DMF (as a solvent) [50] were the most frequently applied conditions. K_2_CO_3_ (as a base) and DMSO (as a solvent) were also reported [51]. Generally, as seen from the literature review, it could be speculated that Ullmann-type reactions could be possible also for chlorinated CNTs. Nevertheless, verification of this hypothesis would require new extensive studies because CNT–Cl as aryl chlorides counterparts could emerge as inert in the catalytic system and hence must have been replaced by bromides or iodides or have been activated by the presence of electron-withdrawing groups (e.g., carboxylic) [52]. We believe that all the above claims would provide a sound subject for the future comprehensive studies. Importantly, the protocol allows for envisaging its direct applicability to other C-sp^2^ allotropes including fullerenes, graphene/graphenoids, nanohorns, quantum dots, and their relatives as well as other functional nanocarbons.

## Figures and Tables

**Table 1:** Characteristics of CNTs used in this study.

Characteristics	Nanocyl™ NC7000	In-House MWCNTs (Aligned Array = ‘Carpet’/’Forest’)	TUBALL™ SWCNTs
Average outer diameter, nm	9.5	60–70	1.6
Average length, µm	1.5	200	>5
Aspect ratio	150	3000	3000
Carbon purity, wt.%	90	98	85
Fe-base catalyst residue, wt.%	<1	5.4	<1.5

CNTs—carbon nanotubes.

**Table 2:** Functionalization degrees (mmol g^−1^ CNTs) of functionalized CNTs as determined from TGA.

Modification	Functionalization Degree [mmol g^−1^ CNTs]			
	Nanocyl NC7000™ MWCNTs		In-House MWCNTs	TUBALL™ SWCNTs
Chlorination by ICl_3_	3.2		2.6	5.0
Halogenation by ICl	0.6		0.4	-
*O*-Arylation	DMF	2.5	1.1	3.5
	Toluene	1.3	0.4	-
	Acetonitrile	1.2	0.4	-
	DMSO	0.9	0.3	-
*C*-Arylation	DMF	2.6	1.3	2.6
	Toluene	1.0	0.8	-
	Acetonitrile	1.2	0.6	-
	DMSO	0.9	0.9	-
*N*-Arylation	DMF	2.7	1.5	2.7
	Toluene	1.3	0.5	-
	Acetonitrile	1.3	0.5	-
	DMSO	1.0	0.5	-

CNTs—carbon nanotubes; TGA—thermogravimetic analysis.

**Table 3:** EDS chemical composition of representative Nanocyl NC7000™ MWCNT samples.

Nanocyl NC7000™ MWCNTs	Element	Atomic Concentration [%]
Chlorinated with ICl_3_	C	95.2 ± 0.5
	Cl	4.6 ± 0.4
	I	0.2 ± 0.05
Chlorinated with ICl	C	93.9 ± 0.1 *
	Cl	3.1 ± 0.3
	I	1.6 ± 0.2
*O*-arylated	C	84.3 ± 0.6
	O	12.6 ± 0.5
	Cl	3.1 ± 0.4
*N*-arylated	C	86.3 ± 0.2
	N	12.5 ± 0.4
	Cl	1.2 ± 0.1
*C*-arylated	C	99.5 ± 0.1
	Cl	0.3 ± 0.1
	I	0.2 ± 0.1

* Al is found due to contamination of commercial MWCNTs with Al_2_O_3_ as the c-CVD catalyst support. EDS Energy-dispersive X-ray spectroscopy.

## Supplementary Materials

The following are available online at https://www.mdpi.com/2079-4991/9/11/1619/s1, Figure S1: Typical HRTEM image of ‘debundled’ SWCNTs functionalized via Ullmann-type reactions, Figure S2: Representative TGA curves recorded under argon for pristine Nanocyl MWCNTs and in-house MWCNTs (A) as well as SWCNTs (B)—all functionalized under optimized conditions (DMF, 120 °C), Figure S3: EDS analysis of MWCNTs: chlorinated using ICl3 (A), chlorinated using ICl (B), *O*-arylated (C), *N*-arylated (D), *C*-arylated (E), Figure S4: Raman spectra of SWCNT: pristine, chlorinated, *N*-arylated, *C*-arylated and *O*-arylated, Figure S5: The Cl 2p energy regions of ICl- and ICl_3_-halogenated MWCNTs. The Cl 2p regions exhibit one highly symmetrical component with its spin-orbit splitting counterpart representing Cl-C bonding at ~200 eV. The spectra were fitted with splitting 1.6 eV and 0.5 area ratio between 1/2 and 3/2 subcomponents, Figure S6: SEM images of the MWCNT forests after chlorination (top (A) and bottom (B) layers) and Ullmann—and Ullmann—type reaction (top (C) and bottom (D) layers, Figure S7: SEM/EDS protocol for MWCNTs forest functionalized using the Ullmann-type reaction—scanned along the nanotubes within the array.
