# Supplementary material for: Ullmann Reactions of Carbon Nanotubes—Advantageous and Unexplored Functionalization toward Tunable Surface Chemistry
Source: Nanomaterials (Basel). 2019 Nov 15;9(11):1619. doi: 10.3390/nano9111619 (PMC6915440; doi:10.3390/nano9111619)
Supplement: Supplementary file 1 [file nanomaterials-09-01619-s001.pdf]

Supplementary Information

for

Ullmann reactions of carbon nanotubes – advantageous and unexplored functionalization toward tunable surface chemistry

A. Kolanowska[1], A.W. Kuziel[1], R. Jędrysiak[1], M. Krzywiecki[2], E. Korczeniewski[3], M. Wiśniewski[3], A.P. Terzyk[3], S. Boncel[1,*]

[1]Department of Organic Chemistry, Bioorganic Chemistry and Biotechnology, Silesian University of Technology, Krzywoustego 4, 44-100 Gliwice, Poland

[2]Institute of Physics – CSE, Silesian University of Technology, Konarskiego 22B, 44-100 Gliwice, Poland

[3]Faculty of Chemistry, Physicochemistry of Carbon Materials Research Group, Nicolaus Copernicus University in Toruń, Gagarin Street 7, 87-100 Toruń, Poland

*Corresponding author. Slawomir Boncel, tel. +48 32 237 12 72, fax +48 32 237 20 94.

E-mail: slawomir.boncel@polsl.pl

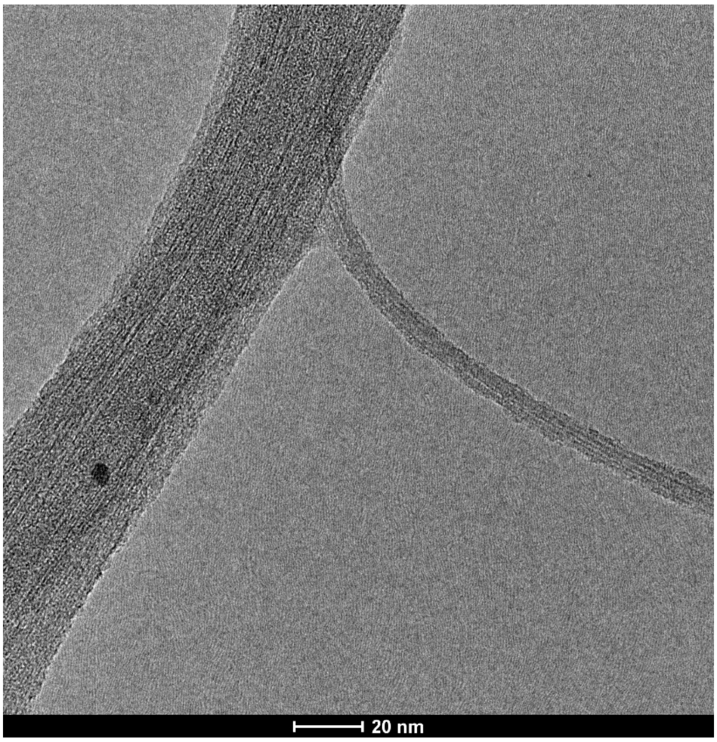

Fig. S1 Typical HRTEM image of 'debundled' SWCNTs functionalized via Ullmann-type reactions

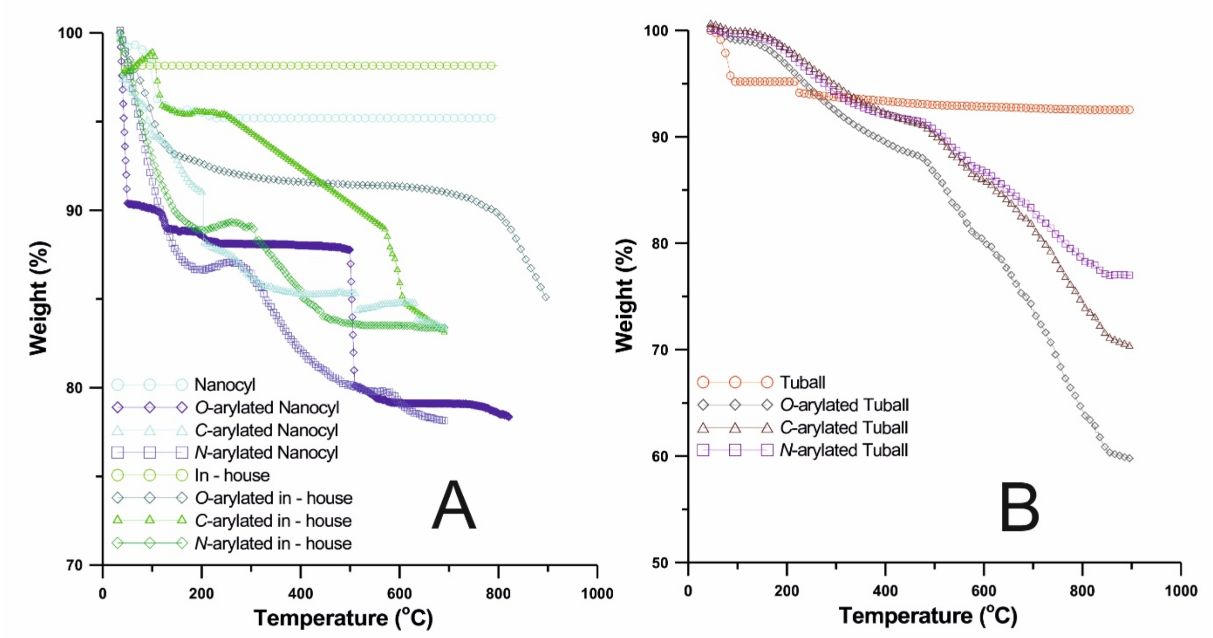

Fig. S2 Representative TGA curves recorded under argon for pristine Nanocyl MWCNTs and in-house MWCNTs (**A**) as well as SWCNTs (**B**) – all functionalized under optimized conditions (DMF, 120 °C)

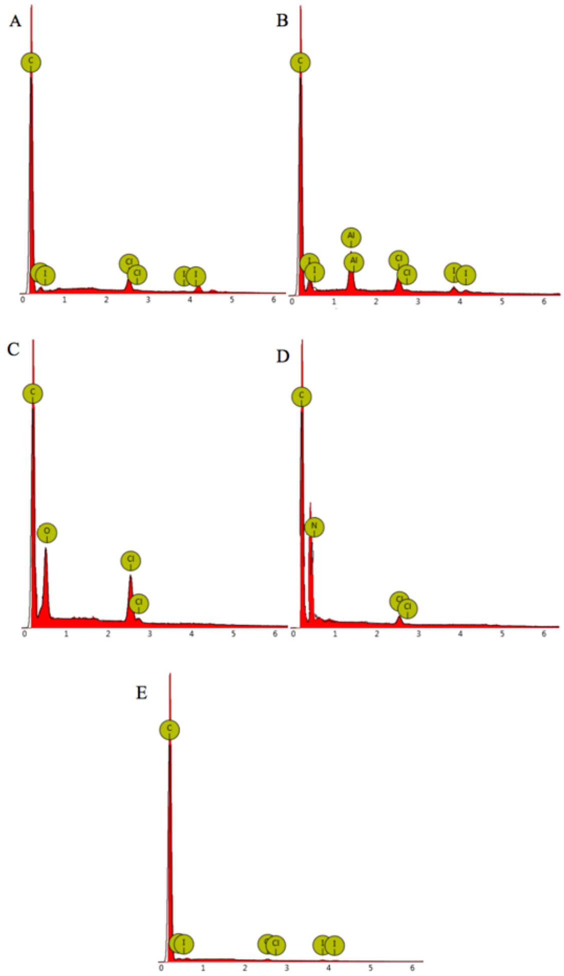

Fig. S3 EDS analysis of MWCNTs: chlorinated using ICl_3 (**A**), chlorinated using ICl (**B**), *O*-arylated (**C**), *N*-arylated (**D**), *C*-arylated (**E**).

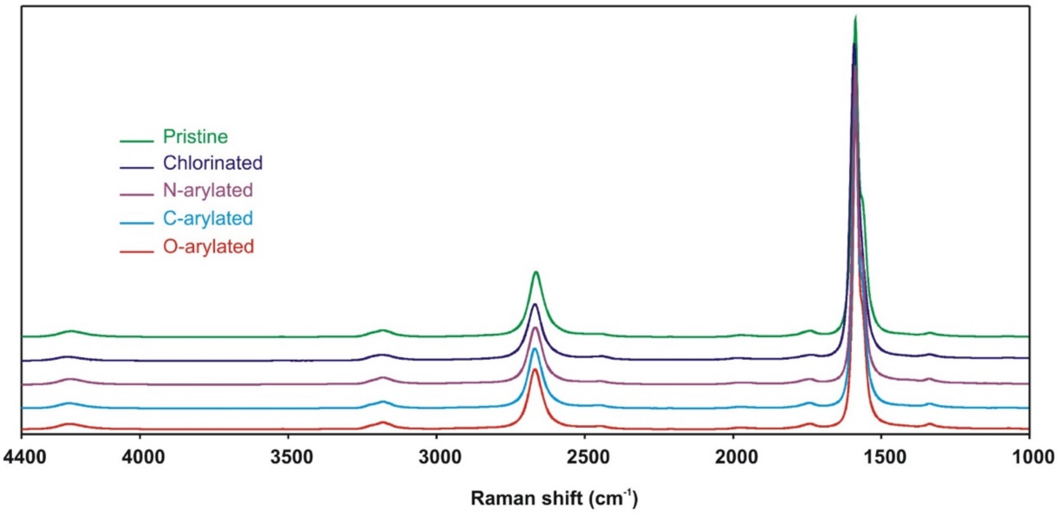

Fig. S4 Raman spectra of SWCNT: pristine, chlorinated, *N*-arylated, *C*-arylated and *O*-arylated.

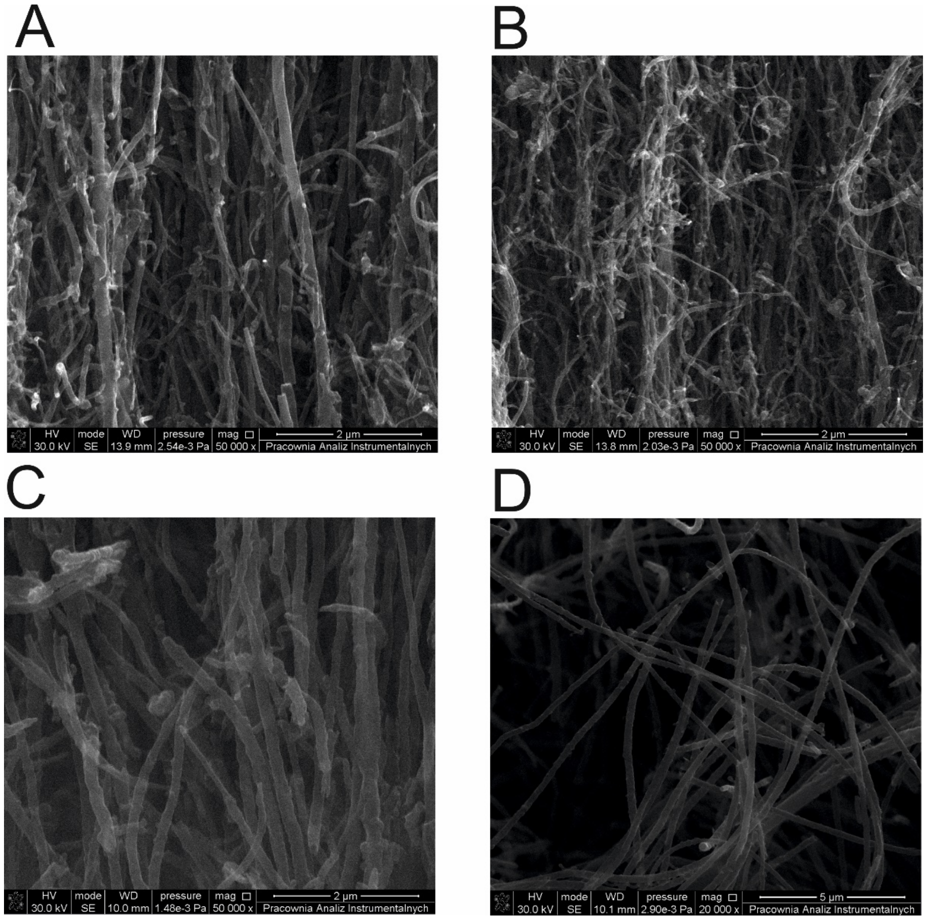

Fig. S5 SEM images of the MWCNT forests after chlorination (top (**A**) and bottom (**B**) layers) and Ullmann – type reaction (top (**C**) and bottom (**D**) layers).

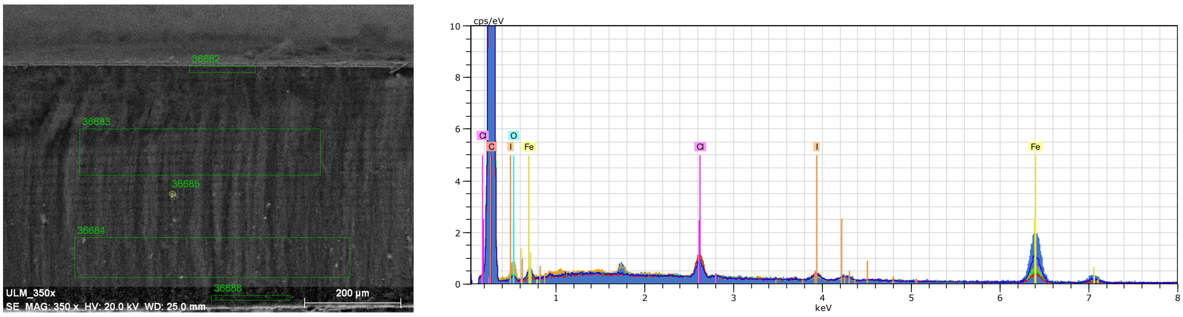

Mass percent (%)

Spectrum	C	O	Cl	Fe	I
36682	90.44	2.49	0.75	5.46	0.86
36683	92.87	3.15	1.05	1.91	1.02
36684	92.32	3.59	0.78	2.53	0.78
36686	88.84	7.35	0.45	2.88	0.48
36685	86.33	2.27	0.55	9.68	1.17
Mean value:	90.16	3.77	0.72	4.49	0.86
Sigma:	2.67	2.07	0.23	3.20	0.26
Sigma mean:	1.19	0.93	0.10	1.43	0.12

Fig. S6 SEM/EDS protocol for MWCNTs forest functionalized using the Ullmann-type reaction – scanned along the nanotubes within the array
